# Impact of Caloric Restriction and Resistance Training on Weight Management, Insulin Sensitivity, and Adipose Tissue Protein Dynamics

**DOI:** 10.1155/omcl/6888340

**Published:** 2026-02-06

**Authors:** Mohammad Mehrtash, Mohsen Salesi, Farhad Daryanoosh, Nader Tanideh, Iman Jamhiri

**Affiliations:** ^1^ Department of Exercise Physiology, Sports Science Faculty, Shahid Bahonar University of Kerman, Kerman, Iran, uk.ac.ir; ^2^ Department of Sport Sciences, Shiraz University, Shiraz, Iran, shirazu.ac.ir; ^3^ Stem Cells Technology Research Center, Shiraz University of Medical Sciences, Shiraz, Iran, sums.ac.ir; ^4^ Molecular Dermatology Research Center, Shiraz University of Medical Sciences, Shiraz, Iran, sums.ac.ir

**Keywords:** caloric restriction, fat oxidation, FSP-27, gene expression, high-fat diet (HFD), insulin resistance, lipid droplet proteins, prilipines 1 and 5, rats, resistance exercise, resistance training

## Abstract

**Background/Aims:**

Obesity and insulin resistance induced by excessive calorie intake remain major health challenges. Caloric restriction (CR) and resistance training (RT) are known strategies to improve metabolic health, but their combined effects on lipid droplet‐associated proteins and metabolic regulators remain unclear. This study aimed to evaluate the impact of CR and RT, alone and in combination (CR + RT), on lipid droplet‐associated proteins and signaling pathways in rats exposed to a high‐fat diet (HFD).

**Methods:**

Fifty male Sprague–Dawley rats were fed HFD for 8 weeks and were then randomly assigned to five groups: HFD, normal‐fat diet (NFD), CR, RT, and CR + RT. Each intervention was performed for 8 weeks following the initial 8‐week HFD induction. Body weight, insulin resistance index (HOMA‐IR), and mRNA expression of perilipin 1 was measured in both adipose and skeletal muscle tissues, whereas perilipin 5, fat‐specific protein 27 (FSP‐27), adipose triglyceride lipase (ATGL), peroxisome proliferator‐activated receptor gamma coactivator 1‐alpha (PGC‐1*α*), sirtuin 1 (SIRT1), and AMP‐activated protein kinase (AMPK) were measured only in skeletal muscle after the subsequent 8‐week intervention period.

**Results:**

At baseline, no significant differences in body weight were observed among the groups (*p*  > 0.05). After 16 weeks, rats in HFD group exhibited the highest body weight (509.8 ± 6.0g), whereas CR + RT group showed the most pronounced reduction (292.2 ± 1.8 g; *p*  < 0.001). Insulin resistance (HOMA‐IR) was significantly elevated in the HFD group (5.55 ± 0.21) compared to all other groups, while the CR + RT group demonstrated the lowest value (1.24 ± 0.05), comparable to the normal diet group (*p*  > 0.05). At the molecular level, CR + RT downregulated perilipin 1 and FSP‐27, while significantly upregulating ATGL, AMPK, SIRT1, and PGC‐1*α* compared to HFD (all *p*  < 0.05).

**Conclusion:**

Combined CR and RT produced superior benefits over either intervention alone, improving insulin sensitivity and lipid metabolism through coordinated regulation of lipid droplet proteins and metabolic signaling pathways. These findings suggest CR+RT as an effective strategy against diet‐induced obesity.

**Summary**

• Caloric restriction (CR) combined with resistance training (RT) produced the greatest reduction in body weight and insulin resistance in high‐fat diet (HFD) rats.
• CR + RT significantly downregulated perilipin 1 and fat‐specific protein 27 (FSP‐27) expression while upregulating adipose triglyceride lipase (ATGL), peroxisome proliferator‐activated receptor gamma coactivator 1‐alpha (PGC‐1*α*), sirtuin 1 (SIRT1), and AMP‐activated protein kinase (AMPK), enhancing lipolysis and mitochondrial function.
• The combination of CR and RT provided superior metabolic benefits compared to either intervention alone.
• This study demonstrates that lifestyle interventions targeting both energy intake and muscle activity can synergistically improve lipid metabolism.
• Findings provide mechanistic insights into obesity prevention strategies by linking exercise and dietary restriction to molecular regulation of lipid droplet proteins. Graphical abstract (Supporting Information Figure S1).

## 1. Introduction

Excessive caloric intake leads to lipid accumulation in adipose and skeletal muscle tissues, causing lipotoxicity, oxidative stress, mitochondrial dysfunction, and insulin resistance. This effect is more pronounced in non‐athletes, while athletes are protected due to higher triglyceride turnover and fatty acid oxidation [1–3].

Lipid droplets are encased by a phospholipid monolayer that includes perilipins, which regulate access of lipases to stored triglycerides [2]. Perilipin 1 is mainly expressed in white adipose tissue, whereas perilipin 5 is abundant in oxidative tissues such as brown fat and skeletal muscles. Hormone‐sensitive lipase (HSL) and adipose triglyceride lipase (ATGL) remain inactive under resting conditions, but upon stimulation, phosphorylation events activate HSL and perilipins, facilitating lipolysis [4]. Although deletion of perilipin 5 in muscle causes insulin resistance and alters lipid droplet composition, the precise mechanisms by which perilipin 5 regulate intramuscular fat remain incompletely understood [5]. Similarly, FSP27 is essential for lipid droplet integrity, and its deficiency is linked to abnormal lipid droplet formation, fatty liver, and insulin resistance [6].

Caloric restriction (CR) has been widely studied for its metabolic benefits and weight loss effects [7]. Sirtuins, particularly SIRT1, play a key role in mediating the beneficial effects of CR by activating mitochondrial biogenesis through proliferator‐activated receptor gamma coactivator 1‐alpha (PGC‐1 *α*) [8, 9]. Alongside this, AMP‐activated protein kinase (AMPK) acts as an energy sensor that phosphorylates and activates PGC‐1*α*, while SIRT1 deacetylates it, forming a regulatory network that enhances mitochondrial function and lipid oxidation [10]. This interplay between AMPK, SIRT1, and PGC‐1*α* is critical for cellular energy homeostasis.

Previous studies have shown that endurance or resistance training (RT) influences perilipin content and lipid metabolism. Ramos et al. demonstrated that endurance training increases perilipin 5 and ATGL levels in adipose tissue [11], while Rinnankoski et al. [10] reported that a high‐fat diet (HFD) elevates perilipin 5 and intramuscular triglycerides, impairing insulin sensitivity, which can be reversed by exercise [12]. Morton et al. [11] further indicated that training increases perilipin 1, perilipin 5, PGC‐1*α*, and FSP27 in skeletal muscle [13]. Similarly, Shepherd et al. [12] showed that RT improves oxidative capacity and intramuscular triglyceride turnover, increasing perilipin expression in both fiber types [14].

While previous studies have examined CR or RT separately, limited research has investigated their combined effects on lipid droplet–associated proteins and signaling pathways under HFD conditions. This study aims to address this gap by examining the effects of CR, RT, and their combination (CR + RT) on mRNA changes in perilipins, ATGL, FSP27, and in the AMPK/SIRT1/PGC‐1*α* axis in adipose and muscle tissues, as well as their role in regulating insulin resistance and metabolic adaptations.

## 2. Methods

### 2.1. Animals and Methods

In the present study, the sample size was 50 male Sprague‐Dawley rats rats aged 8 weeks. Rats were kept in an animal laboratory (Shiraz University) and fed an HFD for 8 weeks, which provided 45% of calories from fat (4.7 kcal/g) and, according to the manufacturer, contained ~24% fat, 24% protein, and 41% carbohydrate, with the remaining fraction consisting of minerals, fiber, and moisture [15]. The number of samples in each group was determined by using the formula for determining the sample size in experimental studies, considering the first type error equal to 0.05, 10 samples in each group. The sample size was estimated using the following formula: *S* = 11.3 (standard deviation) and *D* = 7 (probable accuracy) from previous sources and Z 1.96 from the table of critical values [16]. Using this formula, the calculated sample size per group was *n* ≈ 10 rats.

n=SX2∗Z∝/22D2.

A total of 50 rats were used in this study (*n* = 10 per group). At the beginning of the experiment, four rats were housed per cage; however, during the HFD phase, the number of rats per cage in the HFD group was reduced from four to three to prevent overcrowding and minimize stress‐related behaviors associated with increased body weight, in accordance with animal welfare recommendations. Rats were randomly allocated into five experimental groups using a balanced randomization sequence, with assignments performed by an independent researcher to minimize bias: normal fat diet (NFD), HFD, CR with a restricted diet, RT with a normal fat diet, and both CR + RT.

Cage positions were rotated weekly to minimize environmental confounding, and handling/sampling was standardized to the same time of day to control for circadian effects. CR was applied as a 40% daily reduction in intake for 8 weeks, leading to gradual weight loss and improved metabolic outcomes. Animals were monitored daily for health and activity, with humane endpoints predefined (e.g., > 20% weight loss, infection, or severe lethargy), though none were reached. Exclusion criteria included baseline weight <280 g or evidence of infectious/heart disease, but no such cases were observed.

After the 8‐week experimental period, rats were euthanized by intraperitoneal injection of ketamine (100 mg/kg) and xylazine (10 mg/kg), followed by cervical dislocation to ensure humane death. Fat tissue and soleus (SOL) muscle samples were collected for analysis. All procedures involving animals were conducted in compliance with the ethical standards approved by the Ethics Committee of Shiraz Medical School, which oversees animal welfare (approval number: IR.SUMS.REC.1394.S444). The study adhered to institutional animal care guidelines and the ARRIVE reporting standards. The overall study design, including animal allocation, interventions, and experimental timeline, is shown in Supporting Information Figure S2.

### 2.2. Exercise Protocol and Caloric Restriction

At first, 1 week of familiarization with the laboratory environment was done, and the rats were introduced to the exercise every day for 10–15 min. Rats assigned to RT underwent ladder‐climbing exercises. A load was attached to the base of the tail for each rat. During the first week of training, the load was equivalent to 20% of the rat’s body weight, and it was gradually increased each week, reaching 50% of body weight by the eighth week. Exercises were performed in 3 sets with 5 repetitions. The rest between repetitions was 1 min, and the rest between sets was 2 min, and it continued 2 times a day with an interval of 6 h, 3 days a week, and for 8 weeks [17]. This research calculated CR with a 40% reduction in diet, which continued for 8 weeks. No study protocol was registered prior to the initiation of the experiment [18].

### 2.3. Extraction of Muscle Tissue

Twenty‐four hours after the last training session and following an overnight fast, animals were deeply anesthetized using an intraperitoneal injection of ketamine (75 mg/kg) and xylazine (10 mg/kg). After confirming the absence of reflexes, rats were euthanized by exsanguination via cardiac puncture. Blood was collected using a syringe pre‐coated with EDTA as an anticoagulant. Following blood collection, the chest cavity was fully opened, and the soleus muscle was carefully dissected, rinsed in physiological saline, snap‐frozen in liquid nitrogen, and stored at −80°C for subsequent analyses [12].

### 2.4. Extraction of Adipose Tissue

Adipose tissue samples (epididymal fat pads) were collected immediately after euthanasia. The tissue was snap‐frozen in liquid nitrogen and stored at −80°C until analysis. Gene expression was assessed in a tissue‐specific manner: perilipin 1 was measured in both adipose tissue and skeletal muscle, whereas PLIN5, ATGL, FSP‐27, AMPK, SIRT1, and PGC‐1*α* were analyzed only in skeletal muscle.

For lipid extraction from adipose tissue, the Folch method was used. Approximately 30 mg of tissue was powdered in liquid nitrogen, followed by the addition of 4 mL of chloroform–methanol (2:1, v/v). The homogenate was gently agitated for 2 h and then washed with 2 mL of 0.9% saline. After centrifugation (2000 rpm, 10 min), the lower organic phase was collected and evaporated under vacuum. The dried lipid extract was redissolved in 250 *μ*L of 98% ethanol for subsequent biochemical analysis [11].

### 2.5. RNA Extraction and cDNA Synthesis

RNA extraction was performed using the YTA Total RNA Extraction Mini Kit (Cat No: YT9065) according to the manufacturer’s instructions. Approximately 50 mg of tissue was processed with the column‐based method, and RNA concentration and purity were measured at 260 nm using a Picodrop device, with an average OD of 1.92 indicating acceptable quality. cDNA synthesis was performed from the extracted RNA using the Fermentas kit (K1622), following the manufacturer’s protocol. The synthesized cDNA was stored at −20°C until further analysis. Real‐time polymerase chain reaction (RT‐PCR) was conducted using primers designed by Primer3 software (Table 1), specifically targeting exon–exon junctions to prevent amplification of genomic DNA contamination. This strategy has also been validated in recent methodological studies such as *ExonSurfer* [17]. The *β*_2_‐microglobulin gene was used as an internal control for normalization. PCR amplification was carried out using a SYBR Green Master Mix (Takara), and reactions were performed in triplicate. Cycle threshold (Ct) values were extracted using the real‐time PCR device software. Relative gene expression levels were quantified using the 2^–*ΔΔ*Ct method, which is widely applied in gene expression studies. Positive control reactions containing *β*_2_‐microglobulin primers confirmed the presence of valid cDNA in all samples [19].

**Table 1:** Primer sequences.

Gene	Sense primer (5′‐3′)	Antisense primer sequence (5′‐3′)
PLIN1	GTGGCTCTCAGCTGCATGT	ATTAGCAGCTGTGAACTGGGT
PLIN5	CCATCTTGCCTATCAACACTCT	TGCATATGCTGGATCAGCTC
B2m	CGTGCTTGCCATTCAGAAA	ATATACATCGGTCTCGGTGG
Pgc1*α*	CAGAAGCAGAAAGCAATTGAAGA	GTTTCATTCGACCTGCGTAAAG
FSP27	AAGGCATCATGGCCCACAG	TCTCCACGATTGTGCCATCTTC
ATGL	TGCGCAATCTCTACCGCCTCT	CGAAGTCCATCTCGGTAGCCCT
SIRT1	GCCACCAACACCUCUUCAUTT	AUGAAGAGGUGUUGGUGGCTT
AMPK	TTGCGTGTGCGAAGGAAGAACC	CCGATCTCTGTGGAGTAGCAGTCC

Glucose was also measured by colorimetric enzyme in serum/plasma/whole blood? method with glucose oxidase technology and using a glucose kit. The HOMA formula (HOMA‐IR) was also used to calculate insulin resistance [20].

## 3. Statistical Analysis

Descriptive statistics were calculated as mean ± standard deviation. Normality of distribution was assessed using the Shapiro–Wilk test, and group comparisons were analyzed using one‐way or two‐way ANOVA where appropriate, and Tukey’s honestly significant difference (HSD) post hoc test was applied for pairwise comparisons. Statistical analyses were performed using SPSS software version 23 and GraphPad Prism, with a significance level set at *α* = 0.05 [21].

## 4. Results

**Figure 1:**
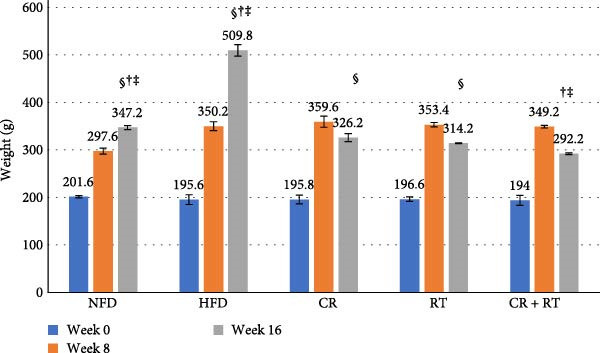
Changes in body weight across groups during the 16‐week intervention. Values are mean ± SD (*n* = 10 per group). One‐way ANOVA with Tukey HSD post‐hoc. At baseline (week 0), no significant differences were observed among groups (*p*  > 0.05). By week 8, rats on a high‐fat diet (HFD) had significantly higher body weights compared to other groups, and by week 16 their weight reached the highest levels (509.80 ± 6.01g, *p*  < 0.001). In contrast, caloric restriction (CR) and resistance training (RT) significantly reduced body weight, while the combined CR + RT intervention produced the greatest reduction (292.20 ± 1.78 g), significantly lower than CR or RT alone (*p*  < 0.001). NFD, normal fat diet group. Statistical significance indicators:  ^∗^: *p*  < 0.05 vs HFD group. #: *p*  < 0.05 vs NFD group. †: *p*  < 0.05 vs CR group. ‡: *p*  < 0.05 vs RT group. §: *p*  < 0.05 vs CR + RT group.

At the beginning of the experiment (week 0), there were no significant differences in the average body weight of rats across all groups (*p*  > 0.05). After 8 weeks of a HFD, rats in the HFD group showed a significant weight gain, reaching an average of 341.92 ± 23.74 g. By week 16, their weight further increased to 509.80 ± 6.01 g (*p*  < 0.001). Similarly, the standard diet group also experienced weight gain, increasing from 201.60 ± 2.40 g at week 0–347.20 ± 4.08 g at week 16. In contrast, the CR group showed a significant reduction in body weight from 359.60 ± 1.51 g at week 8–326.20 ± 2.16 g at week 16. The RT group also demonstrated significant weight loss, dropping from 353.40 ± 4.66 g to 314.20 ± 0.83 g during the same period (*p* ≤ 0.001). Notably, the combined CR + RT group exhibited the most pronounced reduction in weight, decreasing to 292.20 ± 1.78 g at week 16. At this time point, their weight was significantly lower than both the CR (*p* ≤ 0.001) and RT (*p* ≤ 0.001) groups (Figure 1). Throughout the experimental period, no mortality or abnormal clinical signs were observed in any of the groups. Also, weight changes during the study are presented in Supporting Information Figure S3.

**Figure 2:**
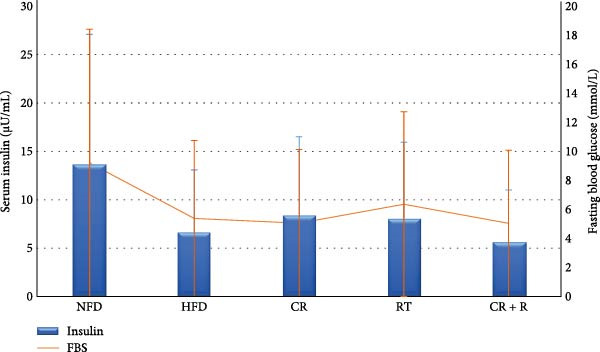
Fasting blood sugar and serum insulin levels in different experimental groups after 16 weeks of intervention. Data are presented as mean ± SD.

**Figure 3:**
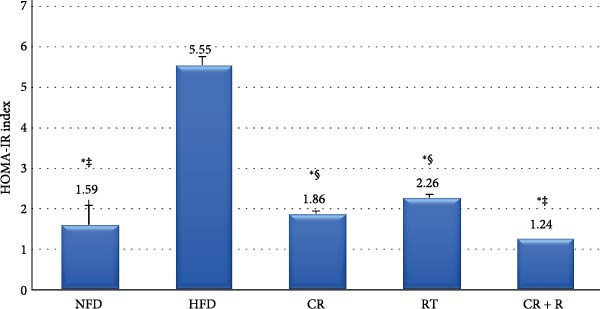
Changes in insulin resistance (HOMA‐IR index) across experimental groups. Data are presented as mean ± SD. Statistical analysis was performed using one‐way ANOVA followed by Tukey HSD post‐hoc test. CR, caloric restriction; HFD, high‐fat diet; NFD, normal‐fat diet; RT, resistance training. Statistical significance indicators:  ^∗^: *p*  < 0.05 vs HFD group. #: *p*  < 0.05 vs NFD group. †: *p*  < 0.05 vs CR group. ‡: *p*  < 0.05 vs RT group. §: *p*  < 0.05 vs CR + RT group.

The information about the changes in insulin and blood sugar in the groups is shown in Figure 2. The study results (Figure 3) indicate that insulin resistance was significantly higher in the HFD group (5.55 ± 0.21) compared to other groups (*p* = 0.001). The HOMA index for the NFD group was (1.59 ± 0.49), significantly lower than the HFD group (*p* = 0.001). The CR group had a HOMA index of (1.86 ± 0.09), significantly lower than the HFD group (*p* = 0.001) but higher than the CR+R group (1.24 ± 0.05, *p* = 0.006). The CR + R group exhibited the lowest HOMA index (1.24 ± 0.05, *p* = 0.001) and showed no significant difference compared to the NFD group (*p* = 0.190). The RT group had a significantly lower index than the HFD group (*p* = 0.001) and higher than the NFD group (*p* = 0.003), while being higher than the CR + R group (*p* = 0.001) and showing no significant difference from the CR group (*p* = 0.124).

The expression of PLIN1 in the adipose tissue of the HFD group (8.94 ± 0.22) was significantly higher than the other groups (*p* = 0.001). No significant difference was observed between the CR group (9.67 ± 0.28) and the TR group (10.12 ± 0.31) in the expression level of plin1 in adipose tissue (*p* = 0.105). Still, It was significantly lower than the HFD group (8.94 ± 0.22, *p* = 0.003) and higher than the CR + RT (10.58 ± 0.25, *p* ≤ 0.001). Compared to other groups, Perilipin 1 gene expression was significantly lower (*p* = 0.001) in the CR + RT (10.85 ± 0.25). The RT group (10.12 ± 0.31) compared to the HFD (8.94 ± 0.22) had a significantly lower expression (*p* = 0.001), but the CR + RT (10.85 ± 0.25) was higher (*p* = 0.003), but it was not statistically different from the CR group (9.67 ± 0.28) alone (*p* = 0.105). According to the results in, there was no significant difference in the expression of the perilipin 1 gene in the muscle tissue between the groups (*p* = 0.129) (Table 2).

**Table 2:** Gene expression.

Gene	Groups	NFD	HFD	CR	RT	CR + R	F	*p*	Partial *η* ^2^
PLIN1 fat (Dct)	mean	11.44 ^∗^§	8.94§	9.67 ^∗^§	10.12 ^∗^§	10.85 ^∗^‡	50.327	≤ 0.001	0.929
	SD	0.22	0.29	0.28	0.31	0.25			
PLIN1 M (Dct)	mean	2.89	2.33	2.80	2.60	2.90	14.363	0.129	0.289
	SD	0.22	0.37	0.20	0.62	0.32			
PLIN5 M (Dct)	mean	9.36 ^∗^†‡§	8.19#†‡§	6.33 ^∗^#	7.99 ^∗^#	5.56 ^∗^#	103.278	≤ 0.001	0.868
	SD	0.38	0.53	0.72	0.82	0.38			
FSP27 (Dct)	mean	7.54 ^∗^	4.25§	6.15§ ^∗^	6.08§ ^∗^	7.61 ^∗^	107.540	≤ 0.001	0.956
	SD	0.46	0.20	0.19	0.22	0.30			
PGC1*α* (Dct)	mean	10.65§	9.93§†‡	9.67§	9.33§	8.55	26.387	≤ 0.001	0.841
	SD	0.27	0.01	0.27	0.27	0.58			
ATGLm (Dct)	mean	5.48§	7.05§ ^∗^	5.57§ ^∗^	5.44§ ^∗^	4.29 ^∗^	17.869	≤ 0.001	0.781
	SD	0.55	0.53	0.52	0.31	0.60			
AMPK (Dct)	mean	5.62§	8.15§ ^∗^	5.60§ ^∗^	5.80§ ^∗^	4.19 ^∗^	36.055	≤ 0.001	0.878
	SD	0.26	0.12	0.37	1.00	0.43			
SIRT1 (Dct)	mean	6.99§	9.19§ ^∗^	6.58§ ^∗^	6.96§ ^∗^	4.69 ^∗^	70.303	0.000	0.934
	SD	0.38	0.40	0.17	0.53	0.53			

*Note*: Table shows the effects of different interventions on gene expression (Plin1, FSP27, PGC‐1*α*, ATGL, AMPK, and SIRT1) in muscle and adipose tissue of rats after 16 weeks. Data are presented as mean ± SD. CR + RT, combined caloric restriction and resistance training.

Abbreviations: CR, caloric restriction; HFD, high‐fat diet group (control); NFD, normal fat diet group; RT, resistance training.

^∗^ *p*  < 0.05 vs HFD group.

#*p*  < 0.05 vs NFD group.

†*p*  < 0.05 vs CR group.

‡*p*  < 0.05 vs RT group.

§*p*  < 0.05vs CR + RT group.

**Figure 4:**
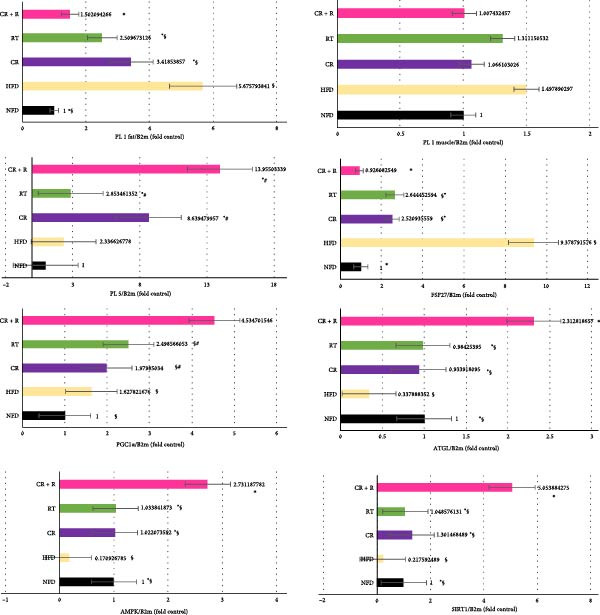
Fold change mRNA expression levels of Plin1 (adipose tissue and skeletal muscle), Plin5, FSP27, PGC‐1*α*, ATGL, AMPK, and SIRT1 genes (skeletal muscle only) in different experimental groups. Data are presented as mean ± SD (*n* = 10 per group). Groups: CR, caloric restriction; CR + RT, caloric restriction + resistance training; HFD, high‐fat diet; NFD, normal‐fat diet; RT, resistance training. Statistical significance indicators:  ^∗^: *p*  < 0.05 vs HFD group. #: *p*  < 0.05 vs NFD group. †: *p*  < 0.05 vs CR group. ‡:*p*  < 0.05 vs RT group. §: *p*  < 0.05 vs CR + RT group.

The expression of perilipin 5 protein was significantly lower in the standard food (9.36 ± 0.38) and high‐calorie food (8.19 ± 0.53) groups compared to other groups (*p* ≥ 0.05). Conversely, it was significantly higher in the rRT group (7.99 ± 0.82), the CR + RT group (5.56 ± 0.38), and the CR group (6.33 ± 0.72) compared to the other groups (*p* ≥ 0.05). However, no significant differences were found among the CR + RT and CR groups (Figure 4).

The FSP27 gene expression in muscles was significantly lower in the CR + R group (7.61 ± 0.30) compared to other groups (*p* = 0.001). The HFD group showed a higher expression (4.25 ± 0.20) than the others (*p* = 0.001). No significant difference was found between the NFD group (7.54 ± 0.46) and the CR+RT group (7.61 ± 0.30) (*p* = 0.994). The CR group (6.15 ± 0.19) did not differ significantly from the RT group (6.08 ± 0.22, *p* = 0.997), but was lower than the HFD group (*p* = 0.001, Figure 4).

The results indicated that neither CR (9.67 ± 0.27, *p* = 0.740) nor RT (9.33 ± 0.27, *p* = 0.074) alone significantly increased PGC1*α* expression compared to the HFD group (9.93 ± 0.01). However, the CR + R group (8.55 ± 0.58, *p* = 0.001) did show increased expression. Both the CR (*p* = 0.001) and RT (*p* ≤ 0.001) groups had significantly higher expression than the NFD group (9.07 ± 0.60), with no significant difference between CR and RT (*p* = 0.528, Figure 4).

For the ATGL gene, expression in the HFD group (7.05 ± 0.53, *p*  ≤ 0.002) was significantly lower than in other groups. The CR + R group (4.29 ± 0.60, *p* = 0.017) had significantly higher expression than the others. No significant differences were found among RT (5.44 ± 0.31, p = 1.000), CR (5.57 ± 0.52, *p* = 0.994), and NFD (5.48 ± 0.55, *p* = 0.999), but all were lower than the CR + R group and higher than the HFD group (Figure 4).

The AMPK gene expression in the HFD group (8.15 ± 0.12, *p* ≤ 0.001) was significantly lower than in other groups. The CR (5.60 ± 0.37, *p* = 1.000) and RT (5.80 ± 1.00, *p* = 0.001) groups did not differ significantly from the NFD group (5.62 ± 0.26), but both had higher expression than the HFD group. The CR + R group (7.05 ± 0.53, *p* ≤ 0.004) showed a significant increase in AMPK expression compared to other groups (Figure 4).

For the SIRT1 gene, expression significantly increased in the CR + R group (4.69 ± 0.53, *p* ≤ 0.001) compared to others, while the HFD group (9.19 ± 0.40, *p* = 0.001) had significantly reduced expression. No significant differences were found between the RT (6.96 ± 0.53, *p* = 1.000) and CR (6.58 ± 0.17, *p* = 0.559) groups compared to the NFD group (6.99 ± 0.38). However, both RT (6.69 ± 0.53, *p* = 0.001) and CR (6.58 ± 0.17, *p* = 0.001) had higher expression than the HFD group (Figure 4).

## 5. Discussion

Our findings showed that 8 weeks of HFD significantly increased body weight, while the CR + RT group displayed the lowest weight at week 16. This reduction was attributable to both lower caloric intake and greater energy expenditure. Previous studies confirm that combining caloric restriction with physical activity enhances negative energy balance and is more effective than caloric restriction alone [22]. Although several investigations, such as Rinnankoski et al. [12], support these findings, the underlying mechanisms go beyond energy intake and expenditure and involve mitochondrial function and lipid regulation.

The HFD group exhibited markedly higher insulin resistance compared to other groups, consistent with evidence linking excessive lipid accumulation in muscle to impaired insulin signaling [23, 24]. This may be mediated by chronic inflammation originating from adipose tissue [25, 26] and mitochondrial dysfunction [27]. By contrast, CR + RT improved insulin sensitivity, likely through reduced lipid deposition and enhanced oxidative capacity. Notably, Pardo et al. [28] reported that CR can improve glucose homeostasis independently of PGC1‐*α*, while other studies emphasize the combined effect of CR and exercise in enhancing insulin sensitivity via mitochondrial pathways [29, 30]. These results highlight that both nutrition and physical activity are critical, but their synergistic effect is particularly beneficial.

Caloric restriction is known to activate sirtuins, especially SIRT‐1, which respond to changes in the NAD^+^/NADH ratio and regulate energy metabolism. In our study, SIRT‐1 expression was lowest in the HFD group and highest in the CR + RT group, consistent with reports that CR and exercise increase SIRT‐1 activity [29, 30]. Exercise also enhances AMPK activity, which acts synergistically with SIRT‐1 to activate PGC1‐*α* and promote mitochondrial biogenesis [33, 34]. Our findings showed upregulation of both AMPK and PGC1‐*α* in the CR + RT group, suggesting that this pathway was a central mechanism mediating improved lipid oxidation and energy balance. This aligns with previous research demonstrating that AMPK and SIRT‐1 act as key effectors of mitochondrial adaptation during CR and exercise [9, 35].

PGC1‐*α*, a master regulator of mitochondrial biogenesis, was markedly elevated in the CR + RT group compared to CR alone. This indicates that exercise amplified the effect of CR on mitochondrial adaptation. Physical activity is known to activate PGC1‐*α* through AMPK, nitric oxide, and MAPK signaling, thereby increasing mitochondrial content and *β*‐oxidation [36–38]. Our results support this, as RT increased PGC1‐*α* expression in oxidative muscle, consistent with studies showing that exercise intensity influences mitochondrial gene expression but that even moderate RT enhances oxidative capacity [39, 40].

FSP27 plays a critical role in lipid droplet morphology and triglyceride storage. In our study, CR + RT decreased FSP27 expression in adipose tissue, consistent with reports linking its downregulation to smaller lipid droplets, enhanced lipolysis, and improved insulin sensitivity [41–43]. Overexpression of FSP27, conversely, has been associated with suppressed mitochondrial *β*‐oxidation and increased fat storage [44]. Thus, reducing FSP27 expression through CR + RT may help mitigate obesity‐associated insulin resistance.

Our data also indicated that CR + RT increased ATGL mRNA expression and reduced perilipin‐1 levels in fat tissue. This is consistent with the mechanism whereby phosphorylated perilipin‐1 releases CGI‐58 to activate ATGL, thereby promoting lipolysis [45, 46]. The elevated ATGL expression observed in our RT groups aligns with previous findings that resistance exercise upregulates ATGL and enhances intra muscle triglyceride turnover [47, 48]. Interestingly, perilipin‐5 expression was increased in skeletal muscle of CR + RT rats despite their lower body weight, suggesting a role in linking lipid droplets to mitochondria and enhancing oxidative metabolism. This interpretation is supported by evidence that perilipin‐5 overexpression can facilitate PGC1‐*α* activation and protect against lipid‐induced insulin resistance [5, 49].

Together, these results highlight that CR + RT exerts its beneficial effects through multiple coordinated mechanisms: improved energy balance, enhanced mitochondrial biogenesis via AMPK–SIRT‐1–PGC1‐*α* signaling, downregulation of FSP27, and favorable modulation of perilipins and ATGL. While our findings provide mechanistic insight into the interplay between diet and exercise, one limitation is the reliance on gene expression data without parallel protein‐level validation. Future studies should incorporate proteomics and phosphorylation analyses to confirm these regulatory effects. Despite this limitation, the results emphasize the translational relevance of combining caloric restriction and resistance training as an effective strategy to combat metabolic dysfunction.

## 6. Conclusion

The present study demonstrates that combining CR with RT enhances metabolic regulation by increasing ATGL mRNA expression while reducing PLIN1 and FSP27 gene expression, thereby promoting lipolysis. Concurrently, CR + RT upregulated SIRT1, AMPK, and PGC1‐*α* signaling, alongside elevated PLIN5 expression, which may facilitate mitochondrial proximity to lipid droplets, stimulate *β*‐oxidation, and improve energy metabolism in skeletal muscle. These adaptations contributed to reductions in body weight and improvements in insulin resistance. Importantly, our findings highlight the synergistic effects of diet and exercise in modulating lipid droplet–associated proteins and mitochondrial biogenesis pathways. Although this study was conducted in rats, the results suggest potential translational value for obesity and metabolic disease management in humans. Future research should confirm these molecular mechanisms at the protein level and evaluate long‐term effects in clinical populations.

**Nomenclature**
AMPK: AMP‐activated protein kinase
ATGL: Adipose triglyceride lipase
CR: Caloric restriction
FSP‐27: Fat‐specific protein 27
HFD: High‐fat diet
HSL: Hormone‐sensitive lipase
IMTG: Intra muscle triglyceride
NFD: Normal‐fat diet
PGC‐1*α*: Peroxisome proliferator‐activated receptor gamma coactivator 1‐alpha
PLIN1: Perilipin 1
PLIN5: Perilipin 5
RT: Resistance training
SIRT1: Sirtuin 1.

## Conflicts of Interest

The authors declare no conflicts of interest.

## Author Contributions

Study concept and design: Mohammad Mehrtash. Acquisition of data: Mohammad Mehrtash. Analysis and interpretation of data: Mohammad Mehrtash and Farhad Daryanoosh. Drafting of the manuscript: Mohammad Mehrtash and Mohsen Salesi. Critical revision of the manuscript: Mohsen Salesi and Nader Tanideh. Statistical analysis: Mohammad Mehrtash. Study supervision: Iman Jamhiri and Nader Tanideh. Scientific consultation and methodological guidance: Farhad Daryanoosh.

## Funding

No funding was received for this manuscript.

## Supporting Information

Additional supporting information can be found online in the Supporting Information section.

## Supporting information


**Supporting Information** Supporting Information Figure 1 presents the graphical abstract summarizing the overall concept and main findings of the study. Supporting Information Figure 2 illustrates the experimental design, including animal allocation, dietary interventions, resistance training protocol, and study timeline. Supporting Information Figure 3 shows body weight changes in all experimental groups from week 1 to week 16.

## Data Availability

The data that support the findings of this study are available from the corresponding author upon reasonable request.
