# Supplementary material for: Impact of Caloric Restriction and Resistance Training on Weight Management, Insulin Sensitivity, and Adipose Tissue Protein Dynamics
Source: Oxid Med Cell Longev. 2026 Feb 6;2026:6888340. doi: 10.1155/omcl/6888340 (PMC12880951; doi:10.1155/omcl/6888340)
Supplement: Supplementary file 1 — Supporting Information Supporting Information Figure 1 presents the graphical abstract summarizing the overall concept and main findings of the study. Supporting Information Figure 2 illustrates the experimental design, including animal allocation, dietary interventions, resistance training protocol, and study timeline. Supporting Information Figure 3 shows body weight changes in all experimental groups from week 1 to week 16. [file OMCL-2026-6888340-s001.docx]

***
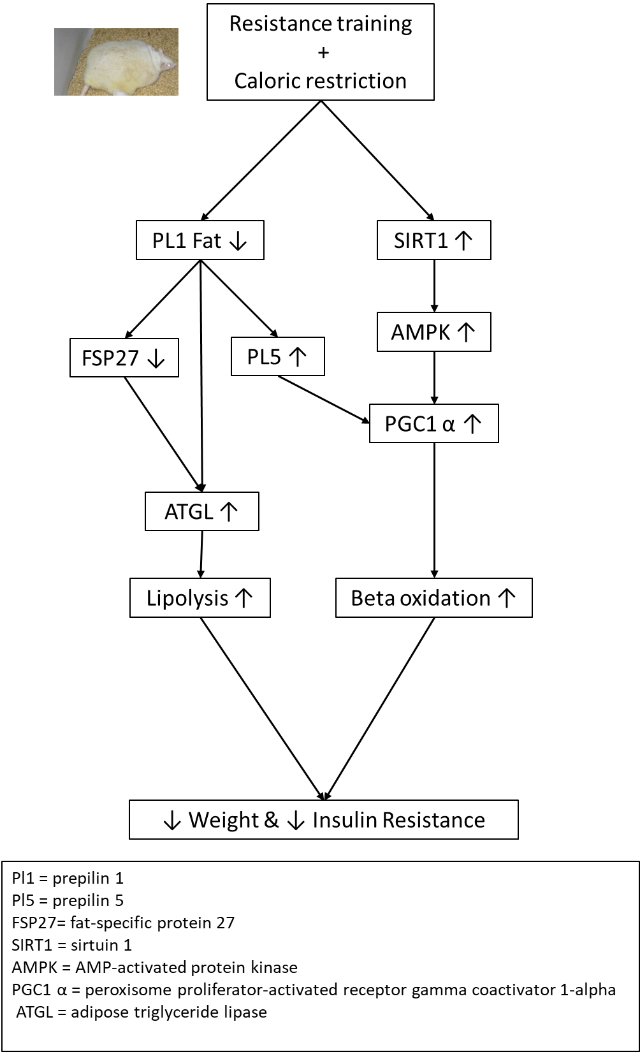
***

supplementary figure 1. Graphical abstract


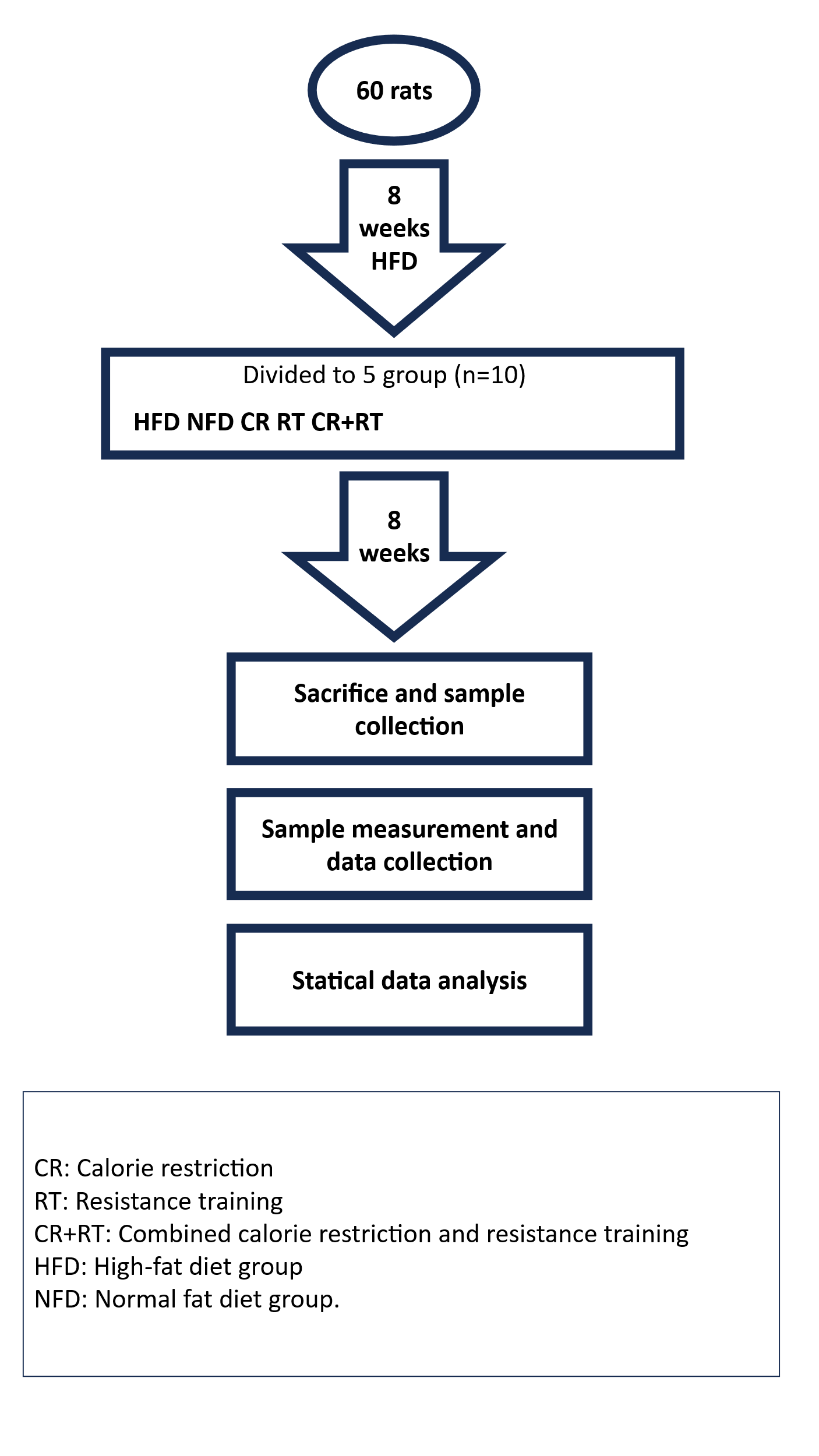


supplementary figure 2. The overall study design

Supplementary Figure 3. Weight Changes week 1 to 16. HFD: High Fat Diet, CR: Caloric Restriction, NFD: Normal Fat Diet, RT: Resistance Training
